# Use of lipolanthionine peptide, a toll-like receptor 2 inhibitor, enhances transdermal delivery efficiency

**DOI:** 10.3892/mmr.2014.2251

**Published:** 2014-05-20

**Authors:** BIN CHEN, DA-LIE LIU, WEN-YAN PAN, XIAO-HUI YANG, JIA-BAO SHOU, JU-HUA WU, QING-LONG MAO, JIA WANG

**Affiliations:** 1 Department of Plastic Surgery, Zhujiang Hospital, Southern Medical University, Guangzhou, Guangdong, P.R. China; 2 Department of Plastic Surgery, Liuzhou Worker’s Hospital, The Fourth Affiliated Hospital of Guangxi Medical University, Liuzhou, Guangxi, P.R. China; 3 Department of Neurosurgery, Liuzhou Worker’s Hospital, The Fourth Affiliated Hospital of Guangxi Medical University, Liuzhou, Guangxi, P.R. China; 4 Department of Rehabilitation Medicine, Liuzhou Worker’s Hospital, The Fourth Affiliated Hospital of Guangxi Medical University, Liuzhou, Guangxi, P.R. China

**Keywords:** toll-like receptor 2, lipolanthionine peptide, retinoic acid, dimethyl sulfoxide, transdermal delivery system

## Abstract

The transdermal delivery system (TDS) is able to obtain a systemic therapeutic effect by administration through the skin, which has low side effects and is able to maintain a sustained blood concentration. However, due to the barrier presented by the stratum corneum, numerous drugs have poor percutaneous permeability. Therefore, the improvement of skin permeability is key to TDS. The main method of promoting transdermal absorption is through the usage of penetration enhancers. Dimethyl sulfoxide (DMSO) is a commonly used penetration enhancer, which has anti-inflammatory analgesic effects and is able to penetrate the skin. Retinoic acid (RA) and lipolanthionine peptide (LP) may also benefit the permeation efficiency of TDS. Therefore, the present study examined the function of DMSO, RA and LP as penetration enhancers in TDS. Firstly, the optimum concentration of DMSO was confirmed by detecting the expression of the LacZ gene *in vitro*. Secondly, different combinations of LP, RA and DMSO were applied to mouse skin to analyze the penetration enhancer combination with the greatest efficacy. All the animals were divided into five groups: The RA + LP + DMSO + pORF-LacZ group, the RA + DMSO + pORF-LacZ group, the LP + DMSO + pORF-LacZ group, the DMSO + pORF-LacZ group and the control group. Skin was soaked in combinations of LP, RA and DMSO for seven days and then the pORF-LacZ plasmids were daubed onto the skin once daily three days. On the 11th day, all the animals were sacrificed by cervical dislocation and the skin and blood samples were collected. The blood samples were used to detect the expression of the LacZ gene by quantitative polymerase chain reaction and the skin samples were used to detect the expression of claudin-4 and zonula occluden-1 (ZO-1) proteins by immunohistochemistry and western blot analysis. The results demonstrated that the combination of LP, RA and DMSO exhibited the greatest transdermal delivery efficiency, which verified that RA and LP were able to increase the penetration effects. Following treatment with LP, the symptoms of dermal edema were relieved and the capillaries contracted, which suggested that LP was a safe and effective penetration enhancer able to reduce the side-effects caused by DMSO. The present study provides a guideline for the synthesis of novel penetration enhancers.

## Introduction

Traditional drug delivery systems include injection and oral administration. The transdermal delivery system (TDS) has been widely used in recent years and is able to exhibit systemic therapeutic effects via administration through the skin. As drugs absorbed via the TDS avoid the first-pass effect in the liver and the degeneration by digestive enzymes in the gastrointestinal tract, TDS enables maintenance of a sustained blood concentration and results in few side effects (1–5). However, due to the barrier presented by the stratum corneum, numerous drugs have poor percutaneous permeability, which means the permeation rate and permeation quantity are not able to meet treatment requirements. Therefore, the improvement of skin permeability is key to TDS. The stratum corneum is the major barrier to transdermal delivery, drugs are absorbed by permeation into the capillaries of the dermis layer through passive diffusion, due to the concentration difference of the drugs on the skin surface and the dermis layer of the skin, and reach the target through the circulation. The main method of promoting transdermal absorption is through the usage of penetration enhancers. These enhancers act by dissolving skin lipids or causing protein denaturation in order to promote drug diffusion in the stratum corneum and increase the rate of transdermal absorption (6–10). Dimethyl sulfoxide (DMSO) is a commonly used penetration enhancer, which has anti-inflammatory analgesic effects, and is able to penetrate the skin and transport drugs into the human body for therapeutic purposes (7,11,12). Retinoic acid (RA) is able to decrease the migration of pigment and reduce the formation of melanin to enhance transdermal delivery (13). Toll-like receptor-2 (TLR2) is able to protect the stratum corneum and tight junctions of the skin, and its inhibitor, lipolanthionine peptide (LP), may benefit permeation efficiency in the TDS (14,15). Thus, the present study aimed to examine the functions of DMSO, RA and LP as penetration enhancers in TDS.

## Materials and methods

### Animals

Mice (Balb/c; 6–8 weeks old) were purchased from Huafukang Biotechnology Ltd (Beijing, China). This study was approved by the Ethics Committee of Guangxi Medical University (Liuzhou, Guangxi).

### Transdermal delivery

The optimum concentration of DMSO (Sigma-Aldrich, St. Louis, MO, USA) was confirmed *in vitro*. The freshly detached skin was fixed in diffusion cells and then 5, 10 and 30% DMSO and saline were used to soak the skin for seven days to increase the penetration capability of the skin. Next, the skin was suspended; there was a spotting compartment and receptor compartment installed separately in both sides of the diffusion area, with the epidermis face mounted toward the spotting compartment and the receptor compartment filled with RPMI-1640 medium during the experiment. The pORF-LacZ plasmids (InvivoGen, San Diego, CA, USA) were added slowly onto the skin and collected from the bottom once a day for three days. The expression of the LacZ gene was detected by quantitative polymerase chain reaction (qPCR) to determine the optimal DMSO concentration. Secondly, combinations of LP, RA (Sigma-Aldrich) and DMSO were applied in mouse skin to analyze the penetration enhancer with the greatest efficacy. The animals were divided into five groups (n=6): The RA + LP + DMSO + pORF-LacZ group, the RA + DMSO + pORF-LacZ group, the LP + DMSO + pORF-LacZ group, the DMSO + pORF-LacZ group and the control group. LP, RA and DMSO were used to soak the skin for seven days, then the pORF-LacZ plasmids were daubed onto the skin for three days, once a day. On the 11th day, all the animals were sacrificed by cervical dislocation following anesthesia with 350 mg/kg of chloralic hydras and then skin and blood samples were collected. The blood samples were used to detect the expression of the LacZ gene by qPCR and the skin samples were used to detect the expression of claudin-4 and zonula occluden-1 (ZO-1) proteins by immunohistochemistry and western blot analysis.

### Histological analysis

All the skin samples were fixed with 10% formaldehyde for 24 h (pH 7.4), then dehydrated and embedded in paraffin. The samples were cut into 5-μm sections. Hematoxylin and eosin (H&E) staining was performed on the sections and observed under an Olympus microscope (CKX31/41; Olympus, Tokyo, Japan).

### qPCR

The total RNA from blood samples was extracted using TRIzol reagent, the primers were synthesized by Invitrogen Life Technologies (Carlsbad, CA, USA) and the primer sequences were as follows: Forward 5′-TTACTGCCGCCTGTTTTGAC-3′ and reverse 5′-GACTGTAGCGGCTGATGTTG-3′ for LacZ. The 3-glyceraldehyde phosphate dehydrogenase (GAPDH) gene was selected as a reference. The reaction consisted of an initial denaturation step at 95°C for 5 min, 40 thermal cycles and an elongation step at 70°C for 7 min. Each of the 40 thermal cycles consisted of a denaturing step at 95°C for 30 sec, an annealing step at 50°C for 30 sec and an elongation step at 70°C for 30 sec. The relative expression of LacZ was calculated according to the formula: 2^−ΔΔCt^. ΔΔCt = [Ct (LacZ) − Ct (GAPDH)]_experimental group_ − [Ct (LacZ) − Ct (GAPDH)]_control group_. Each sample was set in four wells.

### Immunohistochemical staining

The mouse monoclonal antibody against claudin-4 and ZO-1 proteins were purchased from (Sigma-Aldrich) and the antibodies were diluted at 1:500 for the working concentration. The immunohistochemical staining used the streptomycin avidin-catalase method and normal calf serum was used as the negative control. Finally, the sections were developed with 3,3′-diaminobenzidine and counterstained with hematoxylin.

### Western blot analysis

The skin tissues were ground in liquid nitrogen and then the lysis buffer was added. The concentration of proteins was adjusted to 2.5 μg/μl using the bicinchoninic acid assay. Next, the proteins were boiled at 100°C for 5 min and centrifuged at 12,000 × g for 5 min, and 50 μg of protein was loaded onto polyacrylamide gel. Electrophoresis conditions: The stacking gel was set at 80 V for 20 min and the separation gel was set at 100 V. When the bromophenol blue reached the bottom of the gel, the semi-dry electrotransfer was used (30 mA; 90 min). Following inhibition, the gels were incubated with the anti-claudin-4 and ZO-1 antibodies overnight at 4°C, then the membrane was washed with Tris-buffered saline with Tween-20 and incbated with the horseradish peroxidase-labeled mouse anti-rabbit secondary antibody for 30 min. The membrane was developed with enhanced chemiluminescence luminous liquid.

### Statistical analysis

Data are expressed as the mean ± standard deviation (SD) and analyzed with SPSS 17.0 software (SPSS Inc., Chicago, IL, USA). Comparison of the data between the two groups was analyzed using Student’s t-test and data in the groups were analyzed by repeated analysis of variance. P<0.05 was considered to indicate a statistically significant difference.

## Results

### Expression of the LacZ gene

**Figure 1:**
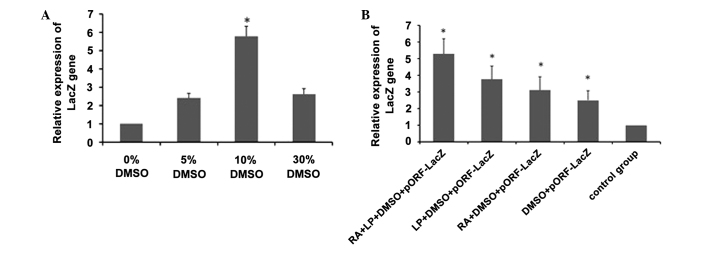
Relative expression of the LacZ gene was determined by quantitative polymerase chain reaction. (A) Expression of the LacZ gene was higher in the 10% DMSO group than that in the 0, 5 and 30% DMSO groups. (B) Expression of the LacZ gene was higher in the RA + LP + DMSO group than that in the LP + DMSO, RA + DMSO, DMSO and control groups. DMSO, dimethyl sulfoxide; RA, retinoic acid; LP, lipolanthionine peptide.

Following the skin being treated with 5, 10 and 30% DMSO, the expression of the LacZ gene was higher in the 10% DMSO group than that in other groups (Fig. 1A), suggesting that 10% DMSO exhibited the optimal transdermal delivery efficiency. Following the skin being treated with RA + LP + 10% DMSO, RA + 10% DMSO or LP + 10% DMSO, the combination of LP, RA and DMSO was demonstrated to have the greatest transdermal delivery efficiency (Fig. 1B), which verified that RA and LP were able to increase the penetration effects of DMSO.

### Pathological changes of the skin

**Figure 2:**
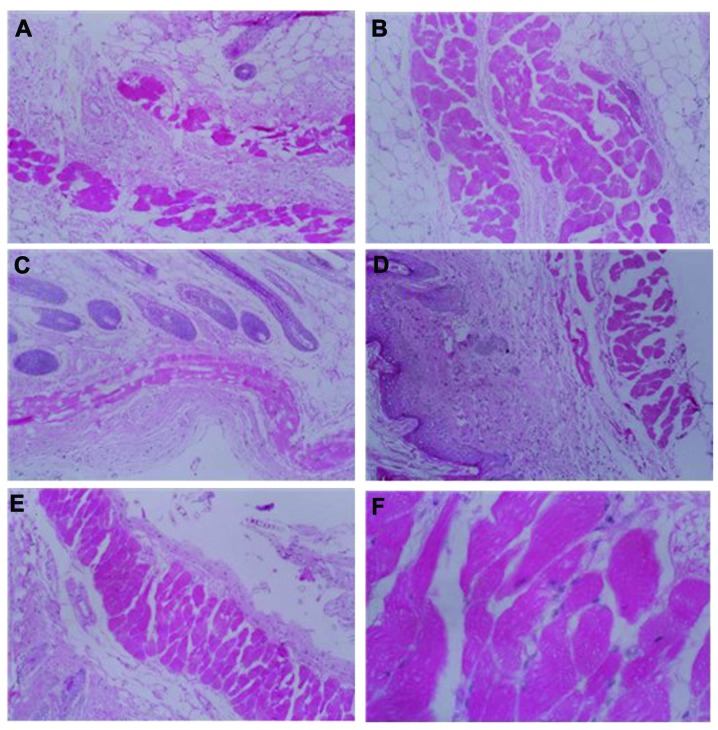
Hematoxylin and eosin staining of skin treated with penetration enhancers in mice. (A) Group RA + LP + DMSO, magnification, ×100; (B) LP + DMSO group, magnification ×100; (C) RA + DMSO group, magnification ×100; (D) DMSO group, magnification ×100; (E) Control group, magnification ×100; (F) Control group, magnification ×400. DMSO, dimethyl sulfoxide; RA, retinoic acid; LP, lipolanthionine peptide.

From H&E staining, the intercellular substance of the stratum corneum was observed to widen and loosen in all the treated groups compared with the control group, and there was no pathological damage to the skin in the control group. Following treatment with DMSO or RA + DMSO, skin papillae, dermal edema, neutrophil aggregation and telangiectasia were observed. While in the RA + LP + DMSO and LP + DMSO groups, the symptoms of dermal edema were relieved and the capillaries were contracted (Fig. 2), which suggested that LP is a safe and effective penetration enhancer as it enhanced the penetration effect and also reduced the side-effects caused by DMSO.

### Expression of claudin-4 and ZO-1 proteins

**Figure 3:**
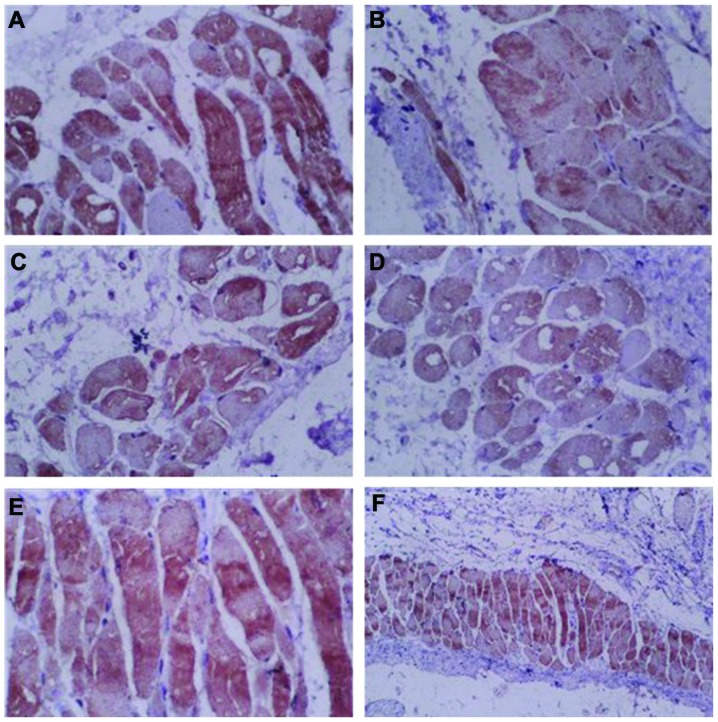
Immunohistochemical staining of claudin-4 proteins in the skin of mice. (A) RA + LP + DMSO group, magnification, ×400; (B) LP + DMSO group, magnification, ×400; (C) RA + DMSO group, magnification, ×400; (D) DMSO group, magnification, ×400; (E) Control group, magnification, ×400; (F) Control group, magnification, ×100. DMSO, dimethyl sulfoxide; RA, retinoic acid; LP, lipolanthionine peptide.

**Figure 4:**
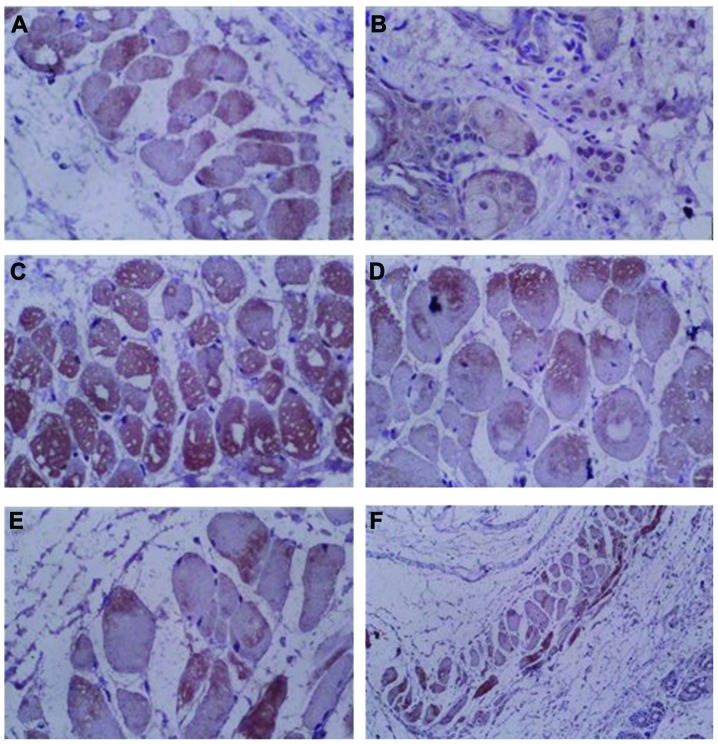
Immunohistochemical staining of ZO-1 proteins in the skin of mice. (A) RA + LP + DMSO group, magnification, ×400; (B) LP + DMSO group, magnification, ×400; (C) RA + DMSO group, magnification, ×400; (D) DMSO group, magnification, ×400; (E) Control group, magnification, ×400; (F) Control group, magnification, ×100. DMSO, dimethyl sulfoxide; RA, retinoic acid; LP, lipolanthionine peptide; ZO, zonula occludens.

In order to observe the expression of tight junction proteins, claudin-4 and ZO-1 proteins were detected by immunohistochemistry and western blot analysis. The immunohistochemical results demonstrated that all groups were positive for claudin-4 protein expression. However, the expression of claudin-4 was lower in the RA + LP + DMSO and LP + DMSO groups compared with that in the control group (Fig. 3), suggesting that the expression of claudin-4 was reduced following treatment with LP. Similarly, the results also demonstrated that ZO-1 proteins were expressed in all the groups and the expression was weaker in the RA + LP + DMSO and LP + DMSO groups (Fig. 4), suggesting that the expression of tight junction proteins was altered following treatment with LP.

**Figure 5:**
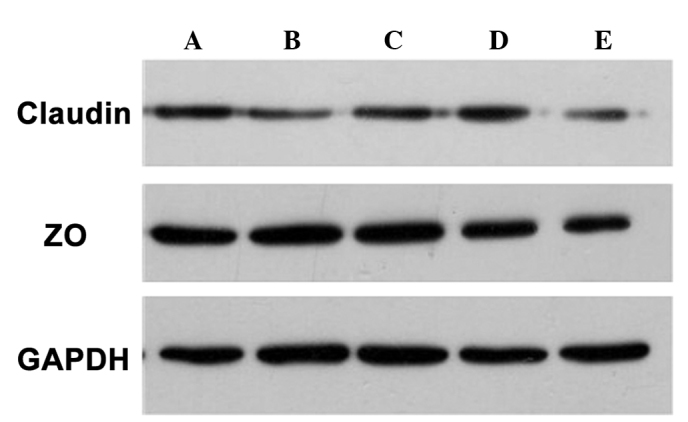
Expression of claudin-4 and ZO-1 proteins in the skin of mice was detected by western blot analysis. (A) Control group; (B) DMSO group; (C) RA + DMSO group; (D) LP + DMSO group; (E) RA + LP + DMSO group. DMSO, dimethyl sulfoxide; RA, retinoic acid; LP, lipolanthionine peptide; ZO, zonula occludens.

Western blot analysis demonstrated that the expression of claudin-4 and ZO-1 proteins was consistent with the immunohistochemical results, that is the expression of claudin-4 and ZO-1 proteins was lower following treatment with penetration enhancers compared with that in the control group (Fig. 5).

## Discussion

The stratum corneum of the skin is composed of lipid bilayers without blood vessels and lymphatic vessels, and is the major barrier to transdermal absorption. Drugs are absorbed by permeation into the capillaries of the dermis layer through passive diffusion, due to the concentration difference of the drugs on the skin surface and in the dermis layer of the skin, and reach the target through the circulation. In addition, the pores, sweat glands and other subsidiary organs are able to absorb a small quantity of the drug. There is a significant difference in transdermal absorption between intact skin and skin without stratum corneum (16–18). Studies have compared the permeability of ferulic acid between intact skin and skin without stratum corneum and revealed that the permeability coefficient of skin without stratum corneum was 12 times that of intact skin (19). Therefore, overcoming the stratum corneum barrier is important for TDS. Previously, DMSO was used as a penetration enhancer; however, DMSO causes skin irritation, and may lead to liver damage and neurological toxicity (7,11,12).

TLRs are newly identified protein molecules, which are a family of type I transmembrane protein receptors containing at least 11 members (20–23). TLRs are capable of pathogen recognition and triggering a series of signal transduction pathways to release various inflammatory mediators and initiate the early innate immune response, which is important for natural immune defense (25,25). In studies of TLRs and the innate immune response, TLR2 has been focused on. TLR2 has a relatively wide range of ligand specificity and is able to recognize a variety of pathogen-associated molecular patters (26). TLR2 is also important in non-infectious tissue injury and tissue repair processes and can combine with TLRs to increase the targeting of ligand binding (27,28). It has been revealed that TLR2 was abundantly expressed in skin inflammation and skin wound healing, which may protect the stratum corneum and tight junctions of the skin (29,30). Therefore, the present study hypothesized that a TLR2 inhibitor may be used as a penetration enhancer to increase transdermal delivery efficiency. LP is a TLR2 inhibitor, which is composed of two amino acids oxidized with cysteine and alanine (15). In the present study, LP was used to treat the skin of mice and the results demonstrated that LP was a safe and effective penetration enhancer and was able to increase the penetration effects, and reduce the damage of the skin caused by DMSO.

The present study compared the functions of LP, RA and DMSO as penetration enhancers in TDS and the results demonstrated that LP, a TLR2 inhibitor, has the strongest transdermal delivery efficiency with no pathological damage to the skin, which has great clinical significance and provides a guideline for the synthesis of novel penetration enhancers.
